# Contribution of Dietary Composition on Water Turnover Rates in Active and Sedentary Men

**DOI:** 10.3390/nu13062124

**Published:** 2021-06-21

**Authors:** Alice E. Disher, Kelly L. Stewart, Aaron J. E. Bach, Ian B. Stewart

**Affiliations:** 1 School of Exercise and Nutrition Sciences, Queensland University of Technology, Brisbane 4059, Australia; a.disher@qut.edu.au (A.E.D.); kelly.stewart@qut.edu.au (K.L.S.); a.bach@griffith.edu.au (A.J.E.B.); 2 National Climate Change Adaptation Research Facility (NCCARF), Griffith University, Gold Coast 4222, Australia

**Keywords:** total body water, hydration, physical activity, nutrition

## Abstract

Body water turnover is a marker of hydration status for measuring total fluid gains and losses over a 24-h period. It can be particularly useful in predicting (and hence, managing) fluid loss in individuals to prevent potential physical, physiological and cognitive declines associated with hypohydration. There is currently limited research investigating the interrelationship of fluid balance, dietary intake and activity level when considering body water turnover. Therefore, this study investigates whether dietary composition and energy expenditure influences body water turnover. In our methodology, thirty-eight males (19 sedentary and 19 physically active) had their total body water and water turnover measured via the isotopic tracer deuterium oxide. Simultaneous tracking of dietary intake (food and fluid) is carried out via dietary recall, and energy expenditure is estimated via accelerometery. Our results show that active participants display a higher energy expenditure, water intake, carbohydrate intake and fibre intake; however, there is no difference in sodium or alcohol intake between the two groups. Relative water turnover in the active group is significantly greater than the sedentary group (Mean Difference (MD) [95% CI] = 17.55 g·kg^−1^·day^−1^ [10.90, 24.19]; *p* = < 0.001; *g*[95% CI] = 1.70 [0.98, 2.48]). A penalised linear regression provides evidence that the fibre intake (*p* = 0.033), water intake (*p* = 0.008), and activity level (*p* = 0.063) predict participants’ relative body water turnover (*R*^2^ *=* 0.585). In conclusion, water turnover is faster in individuals undertaking regular exercise than in their sedentary counterparts, and is, in part, explained by the intake of water from fluid and high-moisture content foods. The nutrient analysis of the participant diets indicates that increased dietary fibre intake is also positively associated with water turnover rates. The water loss between groups also contributes to the differences observed in water turnover; this is partly related to differences in sweat output during increased energy expenditure from physical activity.

## 1. Introduction

Body water turnover refers to the movement and replacement of water through the body, typically over a day, and denotes fluid homeostasis [1]. Therefore, accurate assessment of body water turnover can be particularly useful in predicting (and hence, managing) fluid loss in individuals to prevent potential physical [2], physiological [3,4] and cognitive [5] declines associated with hypohydration [6]. The balance of body water, or fluid, is the difference between total fluid gain (influx) and loss (efflux) [1,7]. The majority of the fluid influx in humans is a result of the food and drink consumed, with a minor contribution from water absorbed by the skin or inspired through breathing [8]. Most of the fluid efflux occurs via urine and ‘insensible’ water loss (i.e., exhaled water vapour, or water lost via the skin, but in the absence of sweating) [1,7]. Minor fluid efflux occurs via human faecal matter [7]. Sweat production can also significantly contribute to an individual’s fluid efflux either in times of heightened physical activity and/or in hot climates [9].

Water loss that occurs because of exercise-induced sweating and respiration (non-renal water losses), has been postulated as an important factor separating the water turnover rates of active and inactive individuals [10,11,12,13]. Some studies observe that as physical activity increases, thermoregulatory sweat output is primarily responsible for increases in water turnover [11,14,15,16]. Fluid efflux in active populations resulting from urinary and sweat losses, respiration, and to a lesser extent, substrate oxidation and carbon dioxide removal, has been shown to exceed that of their sedentary equivalents [17,18]. Thus, increased fluid intake in active individuals would be expected to maintain fluid balance.

Ensuring fluid balance can be achieved by consuming adequate fluid volumes, minimising sweat loss (which may not be possible), and/or through diet—by consuming high moisture content foods or by increasing intake of nutrients that improve water retention or reduce its elimination. Currently, there is a small pool of research that has examined the comparison of fluid balance between active and inactive individuals [10,11,12,14,19,20]; however the contribution of total water intake from both fluid and foods has not been consistently investigated, with just one study of young swimmers that accounted for the moisture content of food [12]. While this study of eight swimmers and six controls identified that water turnover was greater in the swimmers, due to sweat losses, the seven-day weighed dietary records were only assessed for the contribution of total moisture content of the food to daily water intake. The contribution of key nutrients within moisture-containing foods or involved with water retention was not reported.

Thus, this research aims to investigate the relationship between not only energy balance and water turnover, but also the dietary composition and water turnover in both active and sedentary individuals. It was hypothesised that relative body water turnover would be faster in active individuals than their sedentary equivalents, relating to a greater fluid intake and higher overall energy expenditure.

## 2. Materials and Methods

### 2.1. Participants

A convenience sample of 38 healthy males volunteered to participate in this study; 19 were physically active, and 19 were sedentary. To distinguish between the two groups, participants completed the International Physical Activity Questionnaire (IPAQ) [21] and self-reported an activity level >150 min (moderate to vigorous exercise) per week, or <90 min of low to moderate exercise per week for active and sedentary, respectively. In addition, physically active participants were included to attain a maximal oxygen consumption (VO_2max_) value ≥ 60 mL·kg^−1^·min^−1^. Participants reported being weight stable (≤3 kg fluctuation in the previous three months) at the commencement of participation in the study. Each participant gave voluntary and written informed consent to participate in the study, which received ethical approval from the Queensland University of Technology Human Research Ethics Committee.

### 2.2. Procedures

Before the commencement of the trial (minimum two days), a maximal aerobic capacity (VO_2max_) test was performed by all active group participants, in the form of an incremental test to exhaustion on a motorised treadmill, as per standard laboratory procedure [22] and as previously described [23]. Given the sedentary participants had been undertaking <90 min of low to moderate exercise per week, a VO_2max_ test was not required, due to safety concerns, as well as its perceived difficulty by the potential participants, resulting in a barrier to the enrolment of this cohort.

Each participant was studied over seven consecutive days, and the study was undertaken over four months between June and September in Brisbane, Australia, when the mean minimum and maximum daytime temperature was 11.5 ± 1.2 and 23.4 ± 0.8 °C, respectively. Total body water and water turnover rate were determined by water-soluble tracer (deuterium oxide) administration, and its elimination rate throughout the study (as determined by the enrichment of the tracer in daily urine samples). Daily body mass changes were monitored on participants’ home scales using seven consecutive daily measurements. Body mass changes were also monitored using laboratory scales for three measurements, each separated by 72 h (the time between laboratory visits). Each laboratory visit also involved collecting specimens (i.e., blood and urine) for later analysis. Participants were required to attend three testing sessions over seven days (every third day). The first session involved collecting a baseline urine sample before the administration of a deuterium oxide dose. Baseline body composition analysis was performed for the determination of participants’ fat mass and fat-free mass. Walking and running stride length measurements were also taken, to ensure accurate accelerometry tracking. Blood samples were collected via venipuncture from a superficial forearm vein. Following the initial laboratory session, the subsequent two sessions involved discussion surrounding participants’ food diaries and accelerometry data that had been recorded by participants, since the preceding laboratory visit.

During the study, participants were requested to maintain their normal pattern of exercise, eating and drinking, and daily lifestyle. It was, therefore, planned during the recruitment phase that participants did not have any atypical sporting activities or social commitments that would elicit unusual activity levels or eating or drinking (alcohol) behaviours. A seven-day test period was agreed upon that suited ‘usual’ participant activity and dietary habits.

### 2.3. Measurements and Equipment

Total body water was measured using the stable, non-radioactive, non-toxic isotope deuterium oxide, in the form of water (2H_2_O) and followed the methodology outlined by Colley and colleagues [24]. Participants provided a baseline urine sample before consuming an oral bolus equating to 0.05 g·kg^−1^ body mass of 2H_2_O, consumed from a cup through a straw. The pre-dosing baseline urine sample was used to correct for background 2H_2_O concentration values in the post-dose sample. The plastic cup and straw were weighed following oral ingestion of the dose to account for any leftover dose in the cup. This was then subtracted from the weight of the cup, straw, and dose to give the exact amount of dose ingested. A single, mid-stream urine sample was provided six hours later (±10 min). No void was made between the two collections. Participants were fasted at the time of dosing and were asked to abstain from food or fluid during the equilibrium period to ensure equilibration across participants occurred under similar conditions.

The subsequent methodology followed procedures outlined by Wishart [25]. The enrichment of the local tap water, the dose given and the 2H_2_O in the pre-dose and post-dose samples were measured using isotope ratio mass spectrometry (Hydra, PDZ Europa, Crew, UK). Each urine sample was pipetted into 10 mL exetainers (Labco Ltd., Califon, NJ, USA) for analysis. A catalyst, approximately 1 mg of platinum on alumina powder (Sigma Aldrich Inc., St. Louis, MO, USA) contained in a 0.5 mL vial (Chromacol Ltd., Herts, UK), was introduced into the exetainer, ensuring no direct contact with the urine. Each sample was evacuated for 5 min before being filled with 99% hydrogen gas for no more than 2 s. The samples were left at room temperature for a minimum of 72 h to equilibrate the deuterium in the urine sample with the hydrogen gas above it. All reference waters were prepared at the same time and in the same manner. Isotopic enrichments were expressed as the difference per unit volume (%O) from standard mean ocean water. All assays were performed in duplicate with repeat assays in the laboratory, demonstrating coefficients of variance of <2.0% at low enrichment levels and <1.0% for higher values.

Participants reported to the laboratory between 500 and 1000 h after an overnight fast. Nude body mass was measured upon arrival to each laboratory visit and after the first morning void. The scales were accurate to the nearest 0.05 kg (Tanita BWB-600, Wedderburn, Australia). On their first visit to the laboratory, participants were asked to collect a mid-stream urine sample into a specimen cup. Participants thereafter collected a mid-stream urine sample outside of the laboratory, from the second void each morning for the next six days. It was preferable that the sample was the second void of the day, as an earlier sample would be a concentrated overnight sample and not representative of that collection time point. Individual samples were stored together in a polystyrene storage container for each participant until their next laboratory visit.

Whole nude body mass was measured on participants’ home bathroom scales (type and measurement precision varied, including both digital and mechanical varieties) daily, and these values were compared to the laboratory scales when ascertaining daily body mass fluctuations. If participants consumed food or fluid or voided before being weighed, the weight of urine produced, or foodstuffs consumed, before measuring body mass was accounted for when calculating the change in body mass between home scales and laboratory scales. Aside from a total of seven forgotten measures, as instructed, participants mostly (97.4%) recorded their ‘home’ whole nude body mass after their first void, as close to leaving home before travelling to the laboratory as possible; this elicited the most accurate comparison between participants’ home scales and the laboratory scales (r = 0.999). The second void was then collected once the laboratory nude body mass was recorded. Height was measured to the nearest 0.1 cm, with shoes removed in an upright posture and during inhalation. Body Mass Index (BMI) was then calculated.

Body composition was measured on a participant’s first visit via whole-body air displacement plethysmography using the BODPOD body composition system (Life Measurement, Concord, MA, USA) and performed according to the manufacturer’s instructions and recommendations. Participants wore tight-fitting underwear or bike pants and a swimming cap. The procedure involved a volume calibration with and without a 50.008 L metal cylinder. Participants entered the BODPOD and sat inside the anterior chamber (450 L), which was connected to a rear measuring chamber (300 L) via oscillating diaphragms and breathed normally (relaxed tidal breathing). The recommended procedure, consisting of two measurements of body volume (30 s each), was adopted [26]. Occasionally body volumes differed by more than 150 mL, in which case a third measurement was performed. The reported body composition result by the BODPOD instrumentation was the mean of the two (or the two closest) measurements.

A 6 mL blood sample was drawn at each laboratory visit, from a vein in the antecubital fossa, into a serum separating vacutainer to determine serum osmolality as an indicator of acute hydration status. This sample was centrifuged after 20 min (4000 rpm for 10 min) (Universal 320R, Hettich, Germany), and the serum refrigerated (at 6 °C) for later analysis (within one week from collection). Serum osmolality was measured in duplicate using the freezing point depression technique (Osmomat 030, Gonotec, Berlin, Germany).

Urine samples were analysed for specific gravity using a digital refractometer (PAL-10 S, ATAGO, Tokyo, Japan) within 24 h of collection, to measure individuals’ acute hydration status. Each sample was measured in duplicate. A third reading was taken where these two measures differed. The mean value of the two closest measures was taken as the urine specific gravity.

### 2.4. Dietary Tracking

The first laboratory session familiarised participants with the iPhone^®^ application, Easy Diet Diary (Xyris Software Pty Ltd., Brisbane, Australia), including its use and function. Examples of daily intake were shown to participants to improve their understanding of quality reporting methods, and any questions were fielded.

Participants kept a daily record of their dietary intake (both food and fluid consumption). The six-day self-reported diet diary was entered into the iPhone^®^ application (the software used was only compatible with iPhone, and in the instance that a participant owned an Android, a loan iPhone^®^ was provided). Participants were trained in providing portion sizes by using measuring cup reference values and/or their personal kitchen weighing scales where possible. FoodWorks commercial software (Professional Edition, version 7.0, Xyris Software, Brisbane, Australia) was used to determine total daily water intake (moisture content of foods, as well as consuming water and other fluids), as well as daily intake of energy (kilojoules), macronutrients (grams), and specific micronutrients (sodium, potassium in milligrams). The accuracy of reporting was clarified during each laboratory visit by an interview with an Accredited Practising Dietitian, with particular emphasis on reported quantities of fluids and high-moisture-content foods, as well as suspected diary omissions. Participants were asked to refrain from consuming alcohol during the test period and were instructed to continue their habitual intake of caffeine, to not produce significant anomalies in hydration measures.

### 2.5. Physical Activity Tracking

The first laboratory session was also utilised to familiarise participants with the use of their activity trackers: The FitBit^®^ Flex Wireless Activity Tracker (FitBit). The FitBit is lightweight (16 g) and contains a microelectromechanical system (MEMS) triaxial accelerometer. A proprietary algorithm estimates energy expenditure from the triaxial accelerometer (FitBit^®^ Inc., San Francisco, CA, USA). The participants’ height and weight information, along with their age and gender, were entered into the FitBit^®^ user website (www.fitbit.com, accessed on 14 April 2021). According to the manufacturer’s direction, the FitBit^®^ Flex was worn on the non-dominant wrist to obtain a more accurate step count (activities of daily living typically require higher non-step-related use of the dominant wrist) [27].

Participants were asked to take 20 strides at their normal walking pace along an indoor corridor. This was repeated for participants’ typical running pace in the active participant group. Once familiarisation was complete, individual calibration of stride length was undertaken to ensure accurate accelerometery tracking. Calibration involved the measure of distance travelled over 20 average walking stride lengths, as well as 20 average running stride lengths. The total distance was divided by 20, to calculate the average stride length. Every 20 strides were repeated in triplicate, with the average of the three values reported. Stride length was then entered into the user website.

Data from the FitBit^®^ user website (total steps, distance travelled, energy expenditure) was extracted and used to estimate the energy expenditure of participants at 15-min intervals over every 24 h. In conjunction with this data, records of daily activities to account for anomalies in step count data were collected, whereby participants were asked to keep note (written or mental) of unusual activity that might disturb their FitBit^®^ step results (unusually low or high spikes in steps). Examples included: Cycling where physical activity was occurring despite minimal FitBit^®^ activity; driving where minimal physical activity was occurring despite a higher representation in FitBit^®^ activity, due to hand movements at the steering wheel and/or uneven surfaces eliciting hand/wrist movement; or specific tasks that required the FitBit^®^ to be removed altogether, such as ‘no jewellery’ rules in team sport, or water immersion activity. This information was collected through recall discussions with participants during each laboratory visit.

### 2.6. Statistical Analysis

All analyses were performed in R (Version 3.5.3) [28] using the RStudio environment (Version 1.0.143). The analysis was completed in four main parts. First, linear regression was used to determine differences in the descriptive variables of age, height, body mass, BMI, and VO_2max_ between active and sedentary participants. Second, a linear mixed-effects model was used to determine the difference between groups for each individual’s coefficient of variation of the hydration markers (body mass, serum osmolality and urine specific gravity). The models included a random intercept for each individual to account for the repeated measurements across days. Third, linear regression was used to determine differences in total body water, body composition and body water turnover between active and sedentary participants. Models included activity level as a fixed factor and were implemented using the base R function ‘lm’. Hedges’ *g* [29] and 95% confidence intervals (CI) were calculated for the standardised difference between active and sedentary participants using the ‘effsize’ package [30]. A weighted pooled SD was used as the denominator, and a non-central *t*-distribution for the 95% CI. Hedges’ *g* values were interpreted as small 0.2, medium 0.5 and large 0.8 [29]. Finally, a penalised regression model was performed to predict water turnover based on the independent predictor variables. A bivariate (Pearson) correlation was used to identify the variables that shared at least a small association (i.e., *r* > 0.1 or < −0.1) with water turnover. The initial model contained group and the following predictor variables: Fibre, sodium, energy expenditure, carbohydrate and water intake that were standardised using the equation: *y*′ = *y* − $\bar{x}$/*s*, where ‘$\bar{x}$’ is the sample mean, ‘*s*’ the sample standard deviation, and ‘*y*’ the observed value. The least absolute shrinkage and selection operator (Lasso) models were used to identify redundant predictors, by shrinking their coefficients to exactly zero [31], using the R package ‘glmnet’. In comparison to more traditional variable selection methods, namely, step-forward and step-back selection, the Lasso model identifies the best subset of predictors, while accounting for co-correlation between predictors [32]. Potentially important predictors identified via Lasso were fit in a ‘final’ linear mixed-effects model. For all models, the assumption of normality was confirmed via histogram plots of model residuals. Evidence of a statistical effect or difference was accepted at an α level of 0.05.

## 3. Results

### 3.1. Participant Characteristics

Participants’ anthropometric characteristics are as displayed in Table 1. There was statistical evidence of the active group having a lower body mass (mean difference (MD) Hedges’ *g*[95% CI] = −0.72 [−1.39, −0.07]), BMI (*g*[95% CI] = −0.73 [−1.40, −0.08]), and body fat percentage (*g*[95% CI] = −2.00 [−2.78, −1.21]) than the sedentary. There was no evidence of a difference in age, height or lean body mass between the groups.

### 3.2. Hydration Marker Coefficients of Variation

There was no statistical evidence of a difference in the coefficient of variation between groups for home body mass (β ± SE [95% CI] = 0.14 ± 0.12 [−0.10, 0.38]; *t* = 1.16; *p* = 0.251) laboratory body mass (β ± SE [95% CI] = −0.05 ± 0.26 [−0.55, 0.45]; *t* = −0.20; *p* = 0.841), serum osmolality (β ± SE [95% CI] = −0.14 ± 0.55 [−1.21, 0.92]; *t* = −0.26; *p* = 0.794) or urine specific gravity (β ± SE [95% CI] = 0.14 ± 0.08 [−0.02, 0.30]; *t* = 1.75; *p* = 0.084).

There was statistical evidence of an increase in the coefficient of variation over the days in both home body mass (β ± SE [95% CI] = 0.04 ± 0.01 [0.02, 0.06]; *t* = 3.48; *p* < 0.001) and urine specific gravity (β ± SE [95% CI] = 0.04 ± 0.01 [0.02, 0.34]; *t* = 4.99; *p* < 0.001). This outcome is the result of a few individuals displaying no change in their measurements on the first two or three consecutive timepoints producing a zero percent coefficient of variation. While this scenario occurred in the laboratory body mass (β ± SE [95% CI] = 0.01 ± 0.06 [−0.11, 0.14]; *t* = 0.23; *p* = 0.820) and serum osmolality (β ± SE [95% CI] = 0.06 ± 0.14 [−0.21, 0.34]; *t* = 0.46; *p* = 0.651), these zero percent coefficient of variations influenced each day equally.

### 3.3. Energy Intake and Expenditure

Participants’ energy expenditure and nutrient consumption data are displayed in Table 2. There was statistical evidence of the active group having a higher energy expenditure (MD *g*[95% CI] = 1.12 [0.44, 1.84]), intake of water (*g*[95% CI] = 1.21 [0.53, 1.93]), CHO (*g*[95% CI] = 0.81 [0.16, 1.49]) and fibre (*g*[95% CI] = 1.28 [0.60, 2.00]), compared with the sedentary. There was no evidence of a difference in sodium or alcohol intake between the groups.

### 3.4. Water Turnover and Total Body Water

**Figure 1:**
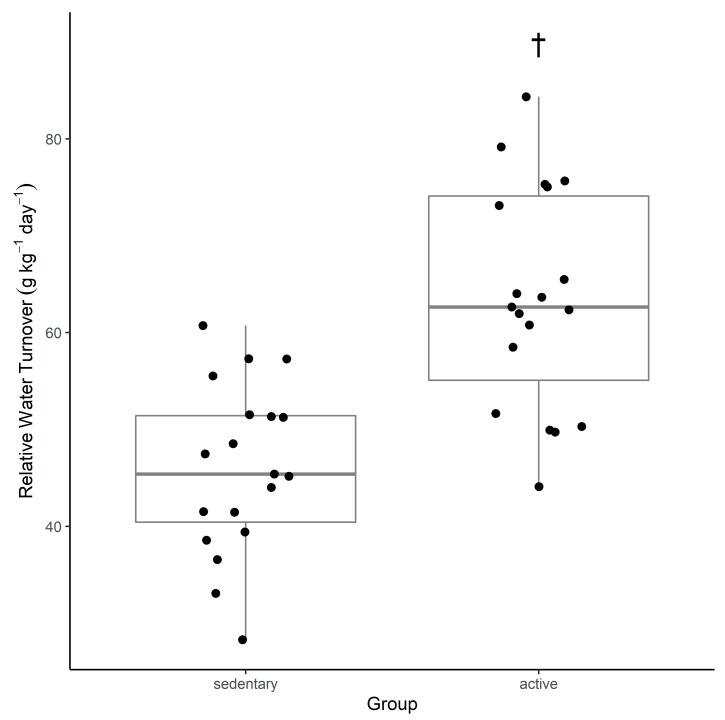
Relative water turnover of active and sedentary participants. † indicates statistical evidence for a difference between groups.

There was statistical evidence the active group had a significantly greater total body water as a percent of body mass (MD *g*[95% CI] = 1.57 [0.86, 2.33]), but not absolute total body water compared with the sedentary. This reflected the fact that active participants tended to have a lower %FM (Table 1). Participants’ relative water turnover is displayed in Table 2 and Figure 1. Relative water turnover in the active group was significantly greater than the sedentary group (MD *g*[95% CI] = 1.70 [0.98, 2.48]).

Penalised regression identified potentially important predictors that were included within a final model. The final model provided evidence that the fibre intake (β ± SE [95% CI] = 4.11 ± 1.85 [0.34, 7.87]; *t* = 2.22; *p* = 0.033), water intake (β ± SE [95% CI] = 4.87 ± 1.74 [1.34, 8.41]; *t* = 2.81; *p* = 0.008), sodium intake (β ± SE [95% CI] = −2.0 ± 1.5 [−5.1, 1.2]; *t =*−1.29; *p* = 0.207), and activity level (β ± SE [95% CI] = 7.38 ± 3.84 [−0.44, 15.21]; *t* = 1.92; *p* = 0.063) predicted the relative body water turnover (F(4,32) = 13.7, *p* < 0.001; R^2^ = 0.585).

## 4. Discussion

The main finding of this study was that the daily water turnover rates were faster in active participants undertaking the regular strenuous activity and with greater energy expenditure, than their sedentary counterparts who undertook little to no planned activity during the assessment period. Absolute TBW content of the active group was not significantly greater than that of the sedentary group; however, relative TBW expressed as a percentage of body mass was greater in the active group. Participants in both groups maintained stable body mass measures across the study duration, and therefore, presumably also maintained stable TBW content, as evidenced by stable hydration measures (U_SG_, S_Osm_ and body mass). This suggests that the exercise-induced energy expenditure among active participants promoted greater water losses (renal or non-renal) daily, leading to faster water turnover. Irrespective of this group effect, water turnover in both groups was significantly influenced by increased water and dietary fibre intake.

This study suggests that active young adult men have a water turnover rate that is faster than that of sedentary men of the same age group (Table 2, Figure 1). A comparison of previous water turnover research and the findings from this study indicate that in the active group, water turnover was observed to be 63.6 ± 11.3 g·kg^−1^·d^−1^ compared with data from other water turnover studies ranging from 47–99.2 g·kg^−1^·d^−1^ [10,11,12,14,15,20,33]. In the sedentary group, water turnover was observed to be 46.0 ± 8.7 g·kg^−1^·d^−1^ compared with data from other water turnover studies ranging from 27.8–53.8 g·kg^−1^·d^−1^ [10,11,12,14,15,20,33]. Previous literature emphasises that there are large daily and individual variations in water turnover rates. Water turnovers exceeding 70 g·kg^−1^·d^−1^ have been seen in individuals undertaking long duration, strenuous activity in harsh environmental conditions, such as firefighters working 12–16 h shifts combatting wildfires across rough terrain in very hot conditions (94.8 g·kg^−1^·d^−1^) [15], and in altitude trekking for an average of 7.5 h (over 17 km) per day (78.7 g·kg^−1^·d^−1^) [19]. The current study values are more representative of regular physical activity, which at times was strenuous (measured by ‘very active minutes’ according to FitBit^®^ data), but not on average reflective of lengthy periods or excessively harsh environmental conditions.

An accelerated water turnover can be explained by larger volumes of fluid intake, as well as by increased body fluid losses. Despite the 24 h human water requirement being linked to anthropometric characteristics (i.e., large individuals with a higher body mass require a greater daily total water intake than smaller individuals) [34], increased fluid intake (via food and fluid) was observed in the active participant group (Table 2). This increase may be explained by additional fluid intake associated with the increase in consumption of overall energy (food), which was required to sustain higher levels of energy expenditure in the active group. Increased exercise-associated energy expenditure relates to an increase in energy intake (to maintain energy balance), which could increase water intake as a by-product of increased calorie-containing fluids, or from the moisture content in foods consumed. Several variables affecting fluid gain depend on physical activity levels: Metabolic water production fluctuates depending on metabolic rate (higher metabolic rates produce more water); and sweat volume can sharply increase in very hot weather or during heavy exercise [1]. Fluid intake can also be enhanced by the presence of greater amounts of food in the gastrointestinal tract, due to central nervous system-mediated mechanisms for eating-induced drinking [35]. Urinary output was not measured in this study, and hence, body fluid losses through urine and other mechanisms can only be estimated. Research is mixed on whether urine output differs between active and sedentary individuals despite increased water turnover rates; however, exercise-induced non-renal water losses (skin, respiration, defaecation) appear to be at least partly responsible for increased water turnover [11,12,14,36].

There is evidence that water turnover is influenced by physical activity—in the longer-term (days to weeks), studies have commonly reported that water turnover is more rapid in physically active individuals compared with their sedentary comparisons [14,19,20]. It has also been found that trained status is significantly correlated to an increase in water turnover [19], likely resulting from increased water intake to assist in thermoregulation when physically active, as well as a physiological adaptation of more efficient sweating, due to an early onset and higher sweat rates. In two distinct studies, Leiper and Maughan [11,14] saw a clear difference of increased fluid losses in their exercising cohort, with one study showing non-renal water losses that were almost three times greater, suggesting to some extent that fluid intake in exercising individuals is greater, to counter exercise-associated increased sweat rates and respiratory fluid losses. Given the enhanced water intakes of the active participants in the present study, this suggests is a requirement to balance greater exercise-induced sweat and respiratory water losses than those of the sedentary participants. These differences in fluid intake between active and sedentary groups may be simply due to differences in habitual fluid consumption; however, it is also possible that differences may be seen due to exercise-induced dehydration stimulating thirst, particularly in the absence of adequate electrolyte replacement from sweat losses [37]. Furthermore, although these studies typically did not directly account for metabolic water production, water intake from foods and fluids, respiration, or sweat production, it is postulated that an increased metabolic rate during exercise elevates these factors, and thus, increases water turnover [20].

The more physically active a participant was (measured via energy expenditure), the greater the water turnover. This is perhaps due not only to increased water intake to replace non-renal losses or an increased fluid intake resulting from increased energy intake, but, in part, because of a metabolic water production derived from substrate oxidation. There is a growing body of evidence supporting the production of metabolic water during the oxidation of exogenous fuel sources in the body [18,38,39,40]; water gains have been reported when glycogen stores are broken down within the muscles and liver [41,42]. Well-trained individuals may have a higher storage capacity for muscle glycogen combined with a higher resting metabolic rate for substrate oxidation. In this situation, if water is endogenously liberated via carbohydrate oxidation, this may explain faster water turnover rates in the active group if this water production does, in fact, contribute to hydration status [19]. As an example, the energy cost of running a marathon for an average 70 kg male is roughly 12,000 kJ (4.18 kJ·kg^−1^·min^−1^) [43]. Estimates of carbohydrate oxidation during this event would indicate that an elite male runner would utilise 400 g of glycogen [44]; given the accepted value of 3 g of water per gram of oxidised glycogen [18], this would result in a 1200 mL endogenous water release.

The link between dietary composition and water turnover remains an under-researched area. This study identified that fibre appears to play an independent role in water turnover. There is very limited literature relating to dietary fibre intake and its relationship to TBW or water turnover; however, from the results of this study, it is apparent that increased fibre intake is positively associated with increased water turnover, independent of participants’ level of physical activity. The human colon can absorb upwards of 5 L of water per day; water and electrolyte absorption in the colon requires the presence of short-chain fatty acids (SCFAs), which are produced by the fermentation of carbohydrates by colonic bacteria [45]. Microbially-fermentable ‘resistant’ starch is responsible for generating SCFAs in the colon due to its indigestibility in the small intestine. SCFAs stimulate blood flow in the colon, and importantly, fluid and electrolyte reabsorption. Resistant starch in an acute dose of around 50 g is documented as a common and effective addition to oral rehydration solutions that are delivered for the management of dehydration caused by severe acute diarrhoea [45,46,47,48] and more recently has shown promising application in treating exercise-induced fluid deficits [49]. The proposed mechanism of action relates to the ability of the colon to absorb sodium against substantial electrochemical gradients, as well as its considerable reserve capacity to absorb fluid [50]. SCFAs are a potent stimulus for the absorption of sodium and water from the colon, hence dietary fibre intake (specifically resistant starch) may play an important role in promoting body water retention, thus slowing water turnover rates [51,52].

While every effort was made to ensure accurate estimation of energy intake and expenditure throughout the study period, recording and reporting bias were expected given the arduous task of participants keeping food and exercise records for a 7-day period. Another limitation of the current study was that 24-h urine collection was not carried out, and hence, fluid loss origins (renal or non-renal) could not be identified.

## 5. Conclusions

This study confirms that water turnover is faster in individuals undertaking regular exercise than in their sedentary counterparts, and is, in part, explained by the intake of water from high-moisture containing foods. The nutrient analysis of the participant diets indicated that increased dietary fibre intake was also positively associated with water turnover rates. The water loss between groups also contributes to the differences observed in water turnover; this is partly related to differences in sweat output during increased energy expenditure from physical activity. Appropriate consideration of the dietary composition of food, in conjunction with fluid intake, may give rise to preventative strategies that could be used to positively manipulate hydration status to abate sweat losses, whether in a clinical, occupational or exercise setting.

## Figures and Tables

**Table 1:** Physical and anthropometric descriptors of study participants.

	Sedentary (*n* = 19)	Active (*n* = 19)	Mean Difference
Characteristic	Mean ± Standard Deviation	Mean ± Standard Deviation	(95% Confidence Interval), *p* Value
Age (yrs)	25.9 ± 3.6	24.7 ± 3.2	−1.2 [−3.42, 1.02], *p* = 0.281
Height (m)	1.80 ± 0.06	1.79 ± 0.06	−0.01 [−0.05, 0.03], *p* = 0.533
Mass (kg) †	87.6 ± 17.1	77.9 ± 7.2	−9.7 [−18.3, −1.0], *p* = 0.029
BMI (kg·m^−2^) †	26.9 ± 4.7	24.3 ± 1.5	−2.58 [−4.86, −0.31], *p* = 0.027
LBM (kg)	63.5 ± 9.0	68.3 ± 5.5	−5.6 [−0.12, 9.71], *p* = 0.056
Fat mass (%) †	24.7 ± 9.9	9.9 ± 3.5	−14.9 [−19.7, −10.0], *p* < 0.001
VO_2_ max (mL·kg^−1^·min^−1^)	-	61.1 ± 2.6	

† indicates statistical evidence for a difference between groups; LBM, lean body mass.

**Table 2:** Mean ± standard deviation participant body water turnover, body composition, and dietary influencing factors of water turnover rates.

	Sedentary (*n* = 19)	Active (*n* = 19)	Mean Difference
Characteristic	Mean ± Standard Deviation	Mean ± Standard Deviation	(95% Confidence Interval), *p* Value
TBW (kg)	46.4 ± 6.8	48.0 ± 3.8	1.8 [−1.9, 5.4], *p* = 0.335
TBW (%) †	53.7 ± 5.6	61.9 ± 4.9	8.4 [4.9, 11.8], *p* < 0.001
WT (L/d) †	4.0 ± 1.0	5.0 ± 1.0	1.0 [0.3, 1.7], *p* = 0.004
WT (g/kg/d) †	46.0 ± 8.7	63.6 ± 11.3	17.6 [10.9, 24.2], *p* < 0.001
Daily H_2_O intake (mL) †	3008 ± 683	4105 ± 1050	1097 [514, 1680], *p* < 0.001
Daily Energy Expenditure (kJ) †	11,412 ± 1769	13,265 ± 1452	1853 [775, 2930], *p* = 0.001
Daily CHO intake (g) †	233 ± 58	296 ± 91	63 [12.9, 113.1], *p* = 0.015
Daily Na intake (mg)	3649 ± 973	3487 ± 1299	−162 [−917, 593], *p* = 0.666
Daily EtOH intake (g)	16.7 ± 17.9	11.9 ± 14.6	−4.8 [−15.5, 5.9], *p* = 0.368
Daily Fibre intake (g) †	20.6 ± 6.9	37.2 ± 16.5	17 [8, 25], *p* < 0.001

† indicates statistical evidence for a difference between groups. TBW, total body water; LBM, lean body mass; WT, body water turnover; EtOH, alcohol.

## Data Availability

The data presented in this study are openly available in Dryad at doi:10.5061/dryad.m37pvmd21.
